# Cloning, characterization and functional analysis of an Alveoline-like protein in the shell of *Pinctada fucata*

**DOI:** 10.1038/s41598-018-29743-6

**Published:** 2018-08-16

**Authors:** Jingjing Kong, Chuang Liu, Tianpeng Wang, Dong Yang, Yi Yan, Yan Chen, Yangjia Liu, Jingliang Huang, Guilan Zheng, Liping Xie, Rongqing Zhang

**Affiliations:** 1 Protein Science Laboratory of the Ministry of Education, School of Life Sciences, School of Life Sciences, Tsinghua University, Beijing, 100084 China; 2 Department of Biotechnology and Biomedicine, Yangtze Delta Region Institute of Tsinghua University, Jiaxing, Zhejiang Province 314006 China

## Abstract

Shell matrix proteins (SMPs) have important functions in biomineralization. In the past decades, the roles of SMPs were gradually revealed. In 2015, our group identified 72 unique SMPs in *Pinctada fucata*, among which Alveoline-like (Alv) protein was reported to have homologous genes in *Pinctada maxima* and *Pinctada margaritifera*. In this study, the full-length cDNA sequence of *Alv* and the functional analysis of Alv protein during shell formation were explored. The deduced protein (Alv), which has a molecular mass of 24.9 kDa and an isoelectric point of 11.34, was characterized, and the functional analyses was explored *in vivo* and *in vitro*. The *Alv* gene has high expression in mantle and could response to notching damage. The functional inhibition of Alv protein *in vivo* by injecting recombinant Alv (rAlv) antibodies destroyed prism structure but accelerated nacre growth. Western blot and immunofluorescence staining showed that native Alv exists in the EDTA-insoluble matrix of both prismatic and nacreous layers and has different distribution patterns in the inner or outer prismatic layer. Taken together, the characterization and functional analyses of matrix protein Alv could expand our understanding of basic matrix proteins and their functions during shell formation.

## Introduction

Biomineralization is a process during which minerals are regulated in an organized way by living organisms. Mollusc shell is one of the most typical objects to study biomineralization^1–3^. For the pearl oyster *Pinctada fucata*, the shell is composed of three layers: the outer periostracum, middle prismatic and inner nacreous layers^4^. In the shell of *P*. *fucata*, organic macromolecules compose only 5% of the weight but play an important role in organizing well-ordered and solid structures^5,6^. Although organic macromolecules consist of proteins, lipids^7^ and polysaccharides, it is thought that matrix proteins are the major components that regulate the biomineralization process, including crystal nucleation, polymorphism, crystal orientation and crystal morphology^6,8–12^. Further up, matrix proteins are thought to potentially contribute to the biomaterials and biomedical fields according to their regulation functions during biomineralization^13^.

Until now, more than 50 matrix proteins have been identified, including Nacrein^4^, Pearlin^14^, Pif^15,16^, KRMP family^17,18^, Prisikin-39^19^, PfN44^20^, Shematrin family^21^, and ACCBP, etc.^22,23^. However, the molecular mechanism of shell formation is still unclear. Traditionally, acidic proteins (pI < 7) are thought to have more functions in the prismatic layer rather than the nacreous layer^12^, and basic proteins (pI > 7) play roles in the nacreous layer other than in the prismatic layer. In fact, most of the reported matrix proteins are acidic or alkalescent proteins, and basic proteins are rarely reported. Until now, only few proteins, that is, the Shematrin family^21^, tyrosinase-like protein 2^24^, KRMPs^17,18^, Prisilkin-39^19^ and PfN23^25^, have all been demonstrated to play important roles in the process of shell formation.

The valine-rich matrix protein, referred as Alveoline-like protein (Alv)^26^, is a typical extreme basic protein with a predicted pI of 11.34. Alv was found via new proteomic approaches with the third abundance in matrix proteins of the prismatic layer^27^. In this study, Alv was proven to have opposite functions in the prismatic and nacreous layers, which is a unique matrix protein with dual roles. To date, only KRMP-3^18^ and MSI7^28^ were suggested to have dual roles in aragonite and calcite formation, which have not been proven *in vivo*. Most matrix proteins only have functions in one layer. For instance, Prisilkin-39^19^, Aspein^29^ and MSI31^30^ play roles in the prismatic layer formation, while Nacrein^31^, Pif97^32^, PfN44^20^, and N40^33^ only have important functions in the nacreous layer.

Rapid-amplification of cDNA ends (RACE) was executed to obtain full-length cDNA sequence of *Alv*. Functional experiments were conducted, including gene expression pattern analysis, notching experiments, and polyclonal antibody injection *in vivo*. Additionally, modified immunofluorescence staining has been applied and brought an interesting discovery. These results offer insights into the functions of Alv during shell formation and shed light on the formation mechanism of prismatic layer.

## Materials and Methods

All methods were executed consistent with approved guidelines. All experiments were confirmed by the Animal Experimental Ethics Committee of Tsinghua University, Beijing, China. The study protocol in the experiments of the animals was approved by the Ethics Committee of National Center for Clinical Laboratories. The 3 R principles about animal experimentation (reduction, replacement, and refinement) were observed strictly. All rabbits used in polyclonal antibodies production were raised under standardized sanitary conditions in the Animal Care Facility at Beijing.

### Sample preparation

The adult pearl oyster, *P*. *fucata* (with shells 5.5–6.5 cm in length and 30–40 g of wet weight at approximately 2 years of age) were cultured in a pearl farm (Zhanjiang, Guangdong Province, China). The oysters were then raised in the laboratory at approximately 20 °C in a fish tank that contained aerated artificial seawater of 3% salinity.

### Primers

Refer to Table 1 for primer details.

**Table 1:** Primers used in this study. F, forward; R, reverse; q, Real-time qPCR.

Primers used for RACE	
Long UPM	CTAATACGACTCACTATAGGGCAAGCAGTGGTATCAACGCAGAGT
Short UPM	CTAATACGACTCACTATAGGGC
NUP	AAGCAGTGGTATCAACGCAGAGT
GSP1	ATGCAGGCGGTTCTGTTTGTCGTTG
NGSP1	GCCTTCGCAGTTCCCCC AAAAGAGA
GSP2	CTAAACCGGATTGGCTCCAAATCCC
NGSP2	TCCCCCAAAGGTCTGTACAGGCCAT
**Primers used to clone Alv to the expression vector pET-28a**	
Alv-p-F	CGCGGATCCGTTCCCCCAAAAGAGATCCAC
Alv-p-R	CCGCTCGAGTTAATGATGCCCGCCTTTTTC
**Primers used for real-time quantitative PCR**	
qAlv-F	GAAGGATACCTGAACCTCGAC
qAlv-R	ACACCCACAGTTTCTACGGAC
qβ-actin-F	CTCCTCACTGAAGCCCCCCTC
qβ-actin-R	ATGGCTGGAATAGGGATTCTGG
qKRMP-F	AAGAAATGTCACCCTTGGGATTGG
qKRMP-R	AATCATCGCCACCATATCCATCG
qNacrein-F	GGCTTTGGCGACGAACCGGA
qNacrein-R	ACACGGGGGAGTGGTCAGGG
**Primers used in in situ hybridization**	
anti-F	CTAATACGACTCACTATAGGGAGAAGGGAAGGATACCTGAACCTCGAC
anti-R	ACACCCACAGT TTCTACGGAC
sense-F	GAAGGATACCTGAACCTCGAC
sense-R	CTAATACGACTCACTATAGGGAGAAGGACACCCACAGTTTCTACGGAC

### Full-length acquisition and analysis of *Alveoline-like* (*Alv*)

The full-length cDNA sequence of the *Alv* was obtained with rapid-amplification of cDNA ends protocol (RACE; SMARTer^TM^ RACE cDNA Amplification Kit, Clontech Laboratories, Inc., Japan). The primers GSP1 and NGSP1 were used for 5′RACE. GSP2 and NGSP2 were applied to 3′RACE. The PCR product was purified using an EasyPure Quick Gel Extraction Kit (TransGen Biotech, China) and was ligated into a pMD18-T vector (TaKaRa, Japan). The recombinant plasmid was sequenced by Beijing Ruibio Biotech Co., Ltd (China). The 5′RACE product sequence and 3′RACE product sequence were then connected together to obtain the full-length cDNA sequence of *Alv*. The *Alv* gene sequence was submitted to the ORF finder website (https://www.ncbi.nlm.nih.gov/orffinder/) to obtain the opening reading frame sequence. The SignalP 4.1 Server website (http://www.cbs.dtu.dk/services/SignalP/) could indicate whether the Alv protein had a signal peptide. The Phyre2 website (http://www.sbg.bio.ic.ac.uk/phyre2/html/page.cgi?id=index) could predict the secondary structure of Alv protein. The Jalview procedure was used to align the amino acid sequences of Alv proteins from different species (*P*. *fucata*, *P*. *margaritifera* and *P*. *maxima*).

### Production of polyclonal antibody of rAlv protein

The production and purification of rAlv protein was produced with hexahistidine (His_6_) tag in the N-terminus but lacking in the signal peptide with primers Alv-p-F and Alv-p-R, and then was applied to 12% SDS-polyacrylamide gel. The target stripe was cut off from the gel, which was stained with Coomassie brilliant blue before. After the standard immunization procedure against New Zealand rabbit, the polyclonal antibodies were raised. The specificity of the antibodies was tested by Western blot against the purified protein with an enhanced HRP-DAB chromogenic substrate kit (TIANGEN Biotech Co., Ltd, China).

### Gene expression pattern analysis by real-time quantitative PCR (qPCR)

There are five different tissues (mantle, foot, gonad, gill and muscle) in *P*. *fucata* in which the *Alv* gene has different expression levels. The pair of primers used for qPCR include qAlv-F/R that was designed for Alv and qβ-actin-F/R which was designed for actin^34^ as reference gene. The qPCR was performed on an ABI PCR amplifier (StepOnePlus^TM^, Life Technologies, USA) following the SYBR® Premix Ex Taq™ (TaKaRa) protocol. The experimental process was as follows: 95 °C, 30 s 95 °C, 5 s; and 60 °C, 30 s for 35 cycles.

### Notching experiment

Notching experiments were executed according to Mount *et al*. with modification^35^. We chose healthy and uniform *P*. *fucata* and cut a “V” shaped breach down to the nacreous layer. The mantle tissue of three pearl oysters was picked at 0 h, 6 h, 12 h, 24 h, 36 h, 48 h, 72 h and 96 h after notching and was placed into liquid nitrogen for future analysis. Total RNA was extracted from mantle samples by TRIzol Reagent (Invitrogen™ TRIzol™, Thermo Scientific, USA). Reverse transcription was executed after checking the integrity of the RNA following the protocol of the PrimeScript^TM^ RT Master Mix (Perfect Real Time; TaKaRa). Then, the qPCR analysis of different notching times was executed. The primers qKRMP-F/R and qNacrein-F/R were used to detect the expression levels of KRMP and Nacrein which were positive controls.

### *In vivo* Alv function interference

Polyclonal antibodies against rAlv were used to inhibit the function of native Alv during shell formation. The purified antibodies were injected into the extrapallial fluid through the byssal foramen at the dosage of 1 µg per g per day using a 0.8 mm × 50 mm syringe needle. Three samples in each group were collected six days later. The shells were separated and washed with Milli-Q water by ultrasonic cleaning. The clean shells were then observed by scanning electron microscope (SEM, 15 kV; FEI Quanta 200, USA) or stained with DyLight® 594-conjucted goat-anti-rabbit antibody for detection of immunofluorescence.

### *In situ* hybridization

The primers anti-F/R were designed to synthesize antisense probes. And the primers of sense-F/R were used to make sense probes. The single-stranded RNA (ssRNA) was produced following the protocol of Promega T7RiboMAX^TM^ with DIG-labeled uracil (Roche Applied Science, USA). Frozen sections of the mantle tissue were obtained using a freezing microtome (LEICA CM1900, Germany). The procedures of hybridization were executed according to the protocol of the Enhanced Sensitive ISH Detection Kit II (AP) (Boster Biological Technology Co., Ltd, China).

### Detection of native Alv in the shell

Cleaned shells of *P*. *fucata* were immersed in 5% sodium hydroxide for 12 h and then washed by diluted water and dried at room temperature (RT). Nacreous layers and prismatic layers were ground using a grinding mill, and then shell powders were sieved with a 100 µm sifter. The calcite prismatic layer and the aragonitic nacreous layer were decalcified with 0.8 M sodium ethylenediaminetetraacetic acid (EDTA; pH 8.0) for 60 h in 4 °C for chromatography with continuous stirring. The supernatant was collected by centrifugation and was then desalted by 3 K ultrafiltration device. The insoluble part was washed with water and was denatured using a denaturing solution (30 mM Tris-HCl, pH 8.0, 1% sodium dodecyl sulfate (SDS), 10 mM dithiothreitol) at 100 °C for 20 min and was then centrifuged for a short duration. The EDTA-insoluble matrix (EISM) and EDTA-soluble matrix (ESM) of the nacreous and prismatic layers were applied to 12% SDS-polyacrylamide gels (SDS-PAGE) which were stained with Coomassie brilliant blue. The existence of the native Alv protein in the ESM and/or EISM of nacre and/or prism was tested by Western blot with the polyclonal antibodies against rAlv as the first antibody (1:1000) and HRP-Goat-anti-Rabbit IgG Fc as the second antibody (1:2000; EarthOx, USA).

Immunofluorescence staining was also employed according to Nudelman *et al*.^36^ with minor modification. First, the shell was cut into pieces of approximately 0.5 cm^2^ with both the prismatic layer and nacreous layer. After being washed and sonicated in distilled water for 10 min, some shell pieces were immersed in 0.5 M EDTA (pH 8.0) for 3 d at 4 °C with gentle shaking. After complete decalcification, some shell pieces were sliced to 30 μm thickness with freezing microtome. Decalcified shell sections, shell squares, and undecalcified shell sections were blocked with 10% bovine serum albumin (BSA) at RT for 2 h. The experimental group and control group were then incubated in PBS containing 10% BSA along with polyclonal antibodies against rAlv (1:50) or preimmune serum (1:50) at RT for 1 h, respectively. Unbounded antibodies were removed by PBS containing Tween 20 (0.05% w/v) of twice for 5 min. Then, shell pieces and sections were incubated with DyLight® 594-conjucted goat-anti-rabbit antibody (1:1000; Agrisera, Sweden) for 30 min at RT in the dark. Unbounded antibodies were removed by PBS containing Tween 20 (0.05% w/v) of twice for 5 min. The shells were then rinsed in water in the dark and observed by inverted laser confocal microscope (Zeiss, LSM780, Germany) within 2 h.

### Statistical analysis

All figures were created using Origin 8 (OriginLab, USA) and Photoshop CC 2015 (Adobe, USA).

### Data and materials availability

GenBank^TM^ Accession No. KR872410.

## Results

### Sequence analyses of the *Alveoline-like* (*Alv*) gene

**Figure 1:**
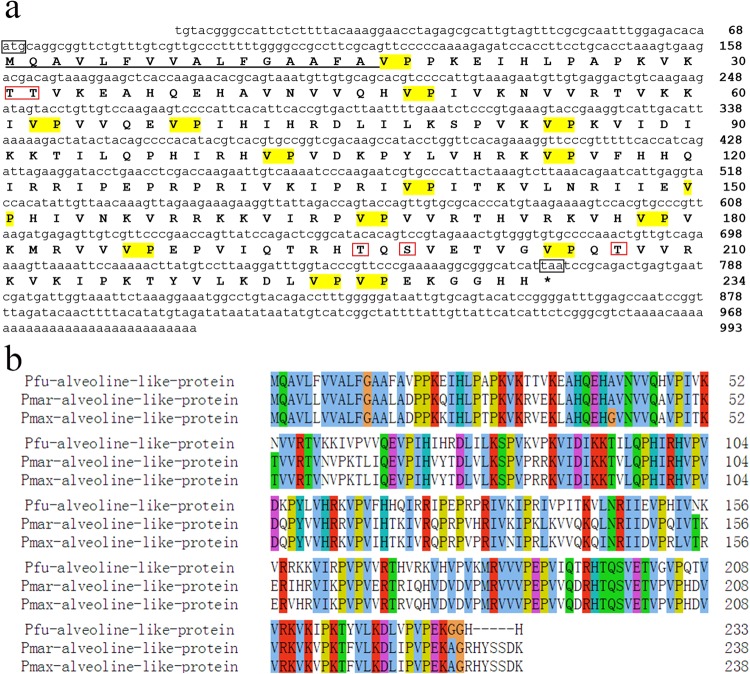
The cDNA sequence information of Alv. (**a**) Nucleotide sequence of *Alveoline-like* (*Alv*) of *P*. *fucata* obtained by RACE. ATG and TAA are the initiation codon and the stop codon, respectively. The sequence before ATG is the 5′-untranslated region (UTR); the sequence after TAA is the 3′- UTR. Translated amino acid sequences of the Alv protein are shown blew nucleotide sequence. Amino acid sequence with underline was predicted as signal peptide of Alv protein. The yellow highlights are VP repeats, and the red boxes are predicted phosphorylation sites. (**b**) Sequence alignment of the Alv protein of *P*. *fucata* (Pfu), *P*. *margaritifera* (Pmar) and *P*. *maxima* (Pmax).

The full-length cDNA sequence of *Alv* was obtained by RACE. We blasted the *Alv* gene with the NCBI database and confirmed the existence of the Alv protein which has homologous amino acid sequence in *P*. *maxima* and *P*. *margaritifera*^26^. Additionally, we confirmed part of the *Alv* gene in the genome of *P*. *fucata* with the transcript ID pfu_aug1.0_89.1_5796-7.t1. The nucleotide sequence of *Alv* obtained by RACE is 993 bp with an open reading frame of 702 bp in length (GenBank^TM^ Accession No. KR872410) (Fig. 1a). It contains a 5′-untranslated sequence of 121-base, a 224-base 3′-untranslated sequence, an ATG in-frame start codon and a TAA in-frame stop codon (Fig. 1a).

The deduced mature Alv protein possesses 217 amino acid residues, and the calculated molecular mass is 24.9 kDa. The Alv protein is characterized with VP repeats. The amino acid composition of Alv is VP-rich, with 23.0% Val and 12.9% Pro, but has low Asp and Glu content which are rich in many other shell matrix proteins (Fig. 1a)^29,37^. In addition, there are 12.0% lysine residues, 8.3% arginine residues, and 7.8% histidine residues, which are basic amino acids (Table 2). In the extrapallial fluid (pH 7.4), these residues are liable to be positively charged; therefore, they are able to interact with negative ions, such as carbonate ions, and negatively charged amino acid residues from acidic proteins^19,37^. The isoelectric point of the Alv protein is 11.34, which is different from most other matrix proteins that are acidic. The Alv protein has five predicted phosphorylation sites, including one Ser and four Thr, which are related to protein functions during biomineralization. The first 16 amino acids are predicted to be a signal peptide, and there is no transmembrane domain predicted in the Alv protein, which is consistent with the secretion character of matrix proteins. The predicted secondary structure of the Alv protein without signal peptide has almost 80% beta strands (Fig. S1), which is unique compared with other proteins. There are Alv homologous proteins in *P*. *maxima* and *P*. *margaritifera* with high conservation, indicating that Alv protein might serve important roles in biomineralization (Fig. 1b). The deduced Alv amino acids were confirmed by comparing with liquid chromatography-mass spectrometry/mass spectrometry (LC-MS/MS) results (Fig. S2a). However, there has been no reports of any known functions.

**Table 2:** The amino acid components of the mature Alv protein.

amino acid	concn, mol%	amino acid	concn, mol%	amino acid	concn, mol%
Alanine	1.4	Glutamic acid	4.1	Lysine	12.0
Arginine	8.3	Glycine	1.4	Methionine	0.5
Asparagine	1.8	Histidine	7.8	Phenylalanine	0.5
Aspartic acid	1.8	Isoleucine	10.1	Proline	12.9
Glutamine	3.7	Leucine	3.7	Serine	0.9
Threonine	5.1	Tyrosine	0.9	Valine	23.0

### Production of the polyclonal antibody anti rAlv protein

The recombinant Alv (rAlv) protein was expressed with a his-tag in the N-terminus by *E*. *coli* (Fig. S2b). The apparent mass of rAlv is around 35 KDa which is larger than the predicted mass about 27.5 KDa. MS analysis has confirmed the existence of Alv protein of *P*. *fucata* in purified rAlv protein. Anti-rAlv antibody was used as first antibody during western blot to test whether the polyclonal antibody work. According to the result of western blot, the specificity of anti-rAlv antibody was confirmed (Fig. S2b).

### *In vivo* investigation of Alv functions in shell formation

**Figure 2:**
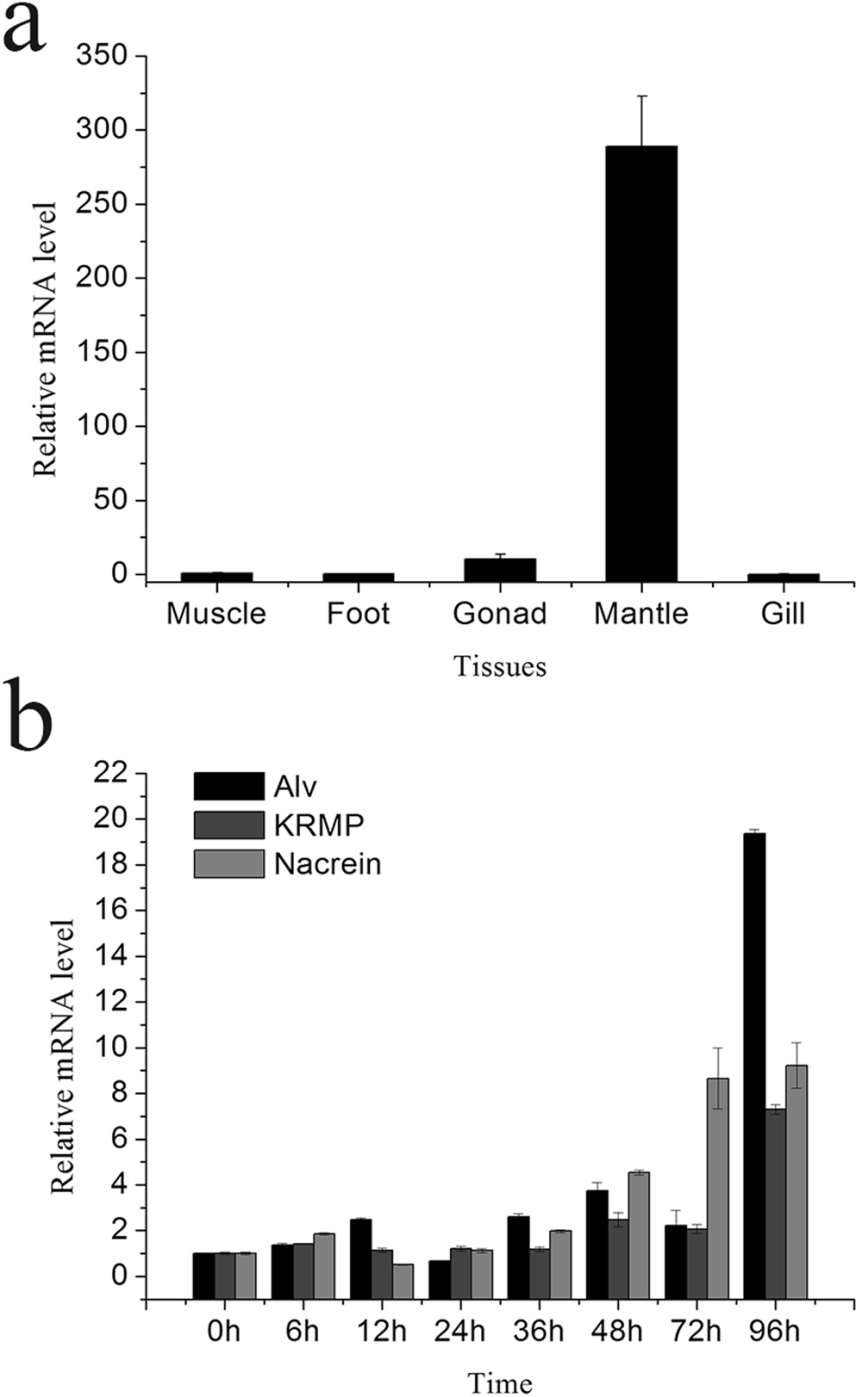
Expression analyses of Alv in *P*. *fucata*. (**a**) Expression level of Alv in different tissues of *P*. *fucata*. (**b**) Alv mRNA expression during the notching experiment.

We analyzed the expression of *Alv* in different tissues of *P*. *fucata* using qPCR (Fig. 2a). The relative expression of *Alv* was detected in the muscle, foot, gonad, mantle and gill, whereas the expression level of *Alv* in the mantle was highest with 287 times higher than expression level in the muscle (the control).

To investigate the Alv functions in shell formation, we conducted assays called “notching experiments”, in which shells were slightly notched to induce shell repair. Gene expression levels of *Alv* at 0 h, 6 h, 12 h, 24 h, 36 h, 48 h, 72 h, and 96 h after the notching treatment are shown in Fig. 2b. We also detected the expression levels of two other matrix proteins, Nacrein and lysine-rich matrix protein 3 (KRMP3), as positive controls^20^. Gene expression levels of *Alv* did not change much until 96 h after notching. *Alv* expression at 96 h reached approximately 19.374 times of the original expression level.

**Figure 3:**
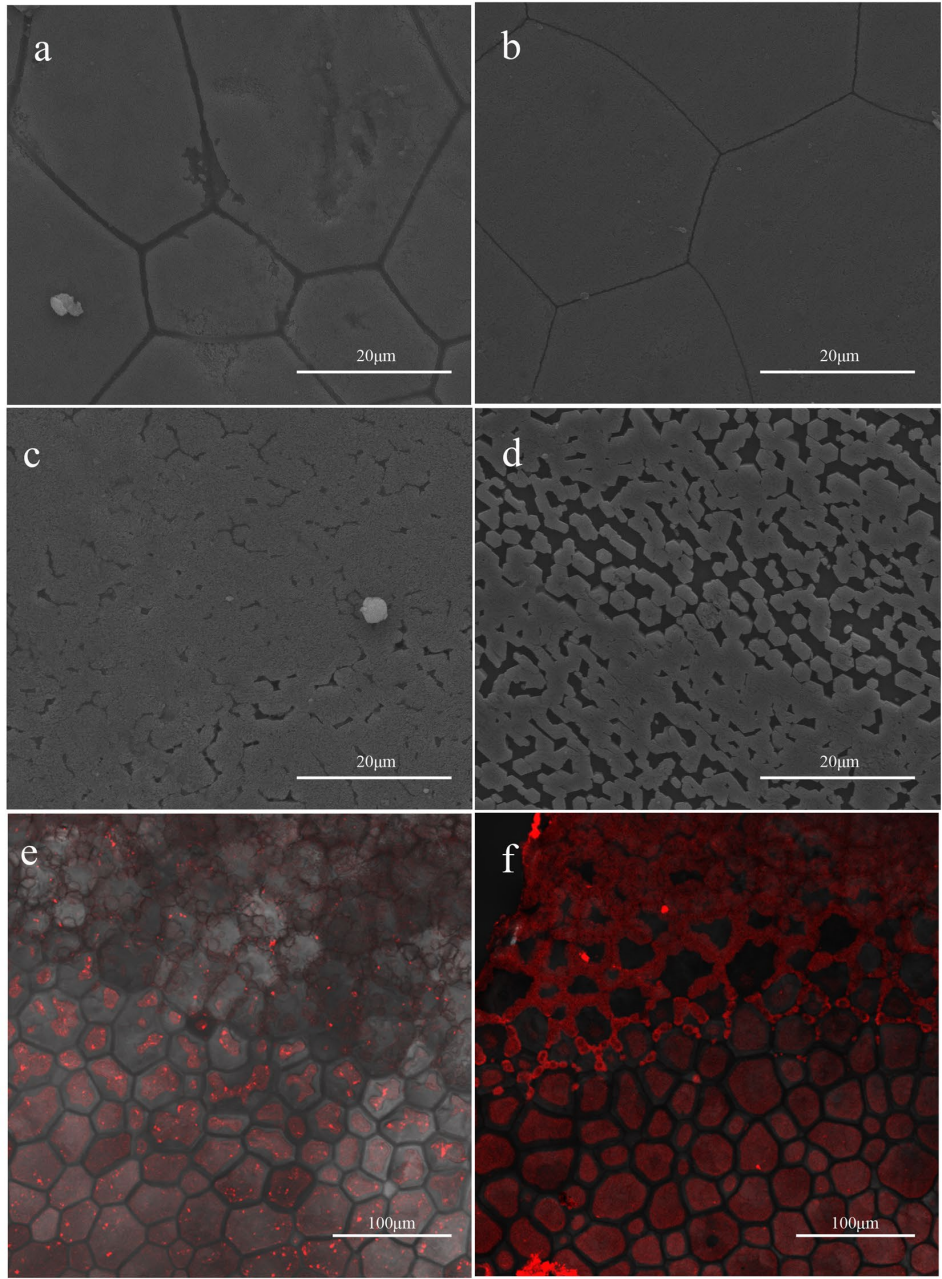
SEM images and immunofluorescence confocal images of shells in the antibody injection experiment. (**a**) SEM image of prismatic layer from the polyclonal antibody-injected group. (**b**) SEM image of prismatic layer from the preimmune serum-injected group. (**c**) SEM image of nacreous layer from the polyclonal antibody-injected group. (**d**) SEM image of nacreous layer from the preimmune serum-injected group. (**e**) Immunofluorescence image of shell from the polyclonal antibody-injected group. (**f**) Immunofluorescence image of shell from the preimmune serum-injected group.

The function of the Alv protein *in vivo* was inhibited by the polyclonal antibody against Alv protein, which was injected into the extrapallial fluid of *P*. *fucata*. As shown in Fig. 3a, the prismatic layer of the shells from the polyclonal antibody-injected group was destroyed and many holes occurred on the surface. On the other side, the nacreous tablets of shell from the polyclonal antibody-injected group overgrew (Fig. 3c). In the meantime, the prismatic and nacreous layers of the preimmune serum-injected group were still intact (Fig. 3b,d). To confirm that the effect of the Alv has been suppressed by polyclonal antibody, we detected the Alv location (Fig. 3e,f) and fluorescence intensity of the antibody-injected group and the preimmune serum-injected group, respectively (Fig. S3). The highest intensity of antibody-injected group is 72, which is less than the highest intensity of control group at 125; in addition, the average intensity of the control group is 40.57, while the average intensity of the antibody-injected group is 8.56 (Fig. S3).

### Expression location details of Alv in *P*. *fucata*

**Figure 4:**
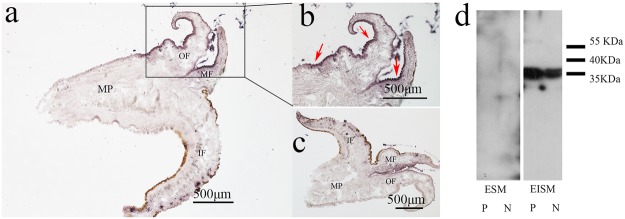
Detection of Alv as a matrix protein. (**a**) Image of mantle with antisense ssRNA probes. (**b**) Enlarged image of box in image a. The red arrows indicate hybridization signals. (**c**) Image of mantle with sense ssRNA probes. OF, outer fold; MF, middle fold; IF, inner fold; and MP, mantle pallial. (**d**) Western blotting analyses of Alv in a matrix protein. ESM, EDTA-soluble matrix; EISM, EDTA-insoluble matrix; P, prismatic layer; N, nacreous layer. Please refer to Fig. S5a,b to get the primary blot pictures.

To explore the precise expression sites of *Alv* mRNA in the mantle, we conducted *in situ* hybridization with sense and antisense ssRNA probes of *Alv*, in which the sense ssRNA probe was used as a negative control. We found that *Alv* mRNA was located in the outer epithelial cells of the outer fold (OF) and outer epithelial cells of the middle fold (MF); no hybridization signals were found in the inner fold (IF) and mantle pallial (MP) (Fig. 4a,b). There are no hybridization signals in negative control group (Fig. 4c).

To investigate whether the Alv protein exists in the prismatic and/or nacreous layer of *P*. *fucata*, matrix proteins were extracted from two layers, respectively. The SDS-PAGE observations of the EISM (EDTA-insoluble matrix) and ESM (EDTA-soluble matrix) matrix proteins from both layers (Fig. S4) were transferred to the PVDF membrane and western blotting followed. The gel bands indicated different compositions of the EISM and ESM matrix proteins from different shell layers (Fig. S4). Western blot results showed that native Alv was detected in the EISM extracted from both the prismatic and nacreous layers but not in the ESM from the two layers (Fig. 4d), which was consistent with the previous study in proteomic analysis of *P*. *fucata*^27^. What’s more, the biochemical location pattern of Alv is consistent with the previous gene expression analyses by qPCR in different tissues. Additionally, the molecular weight of native Alv is almost 10 kDa larger than the predicted molecular weight of Alv, which corresponds to the post-translation modification of Alv in *P*. *fucata*.

**Figure 5:**
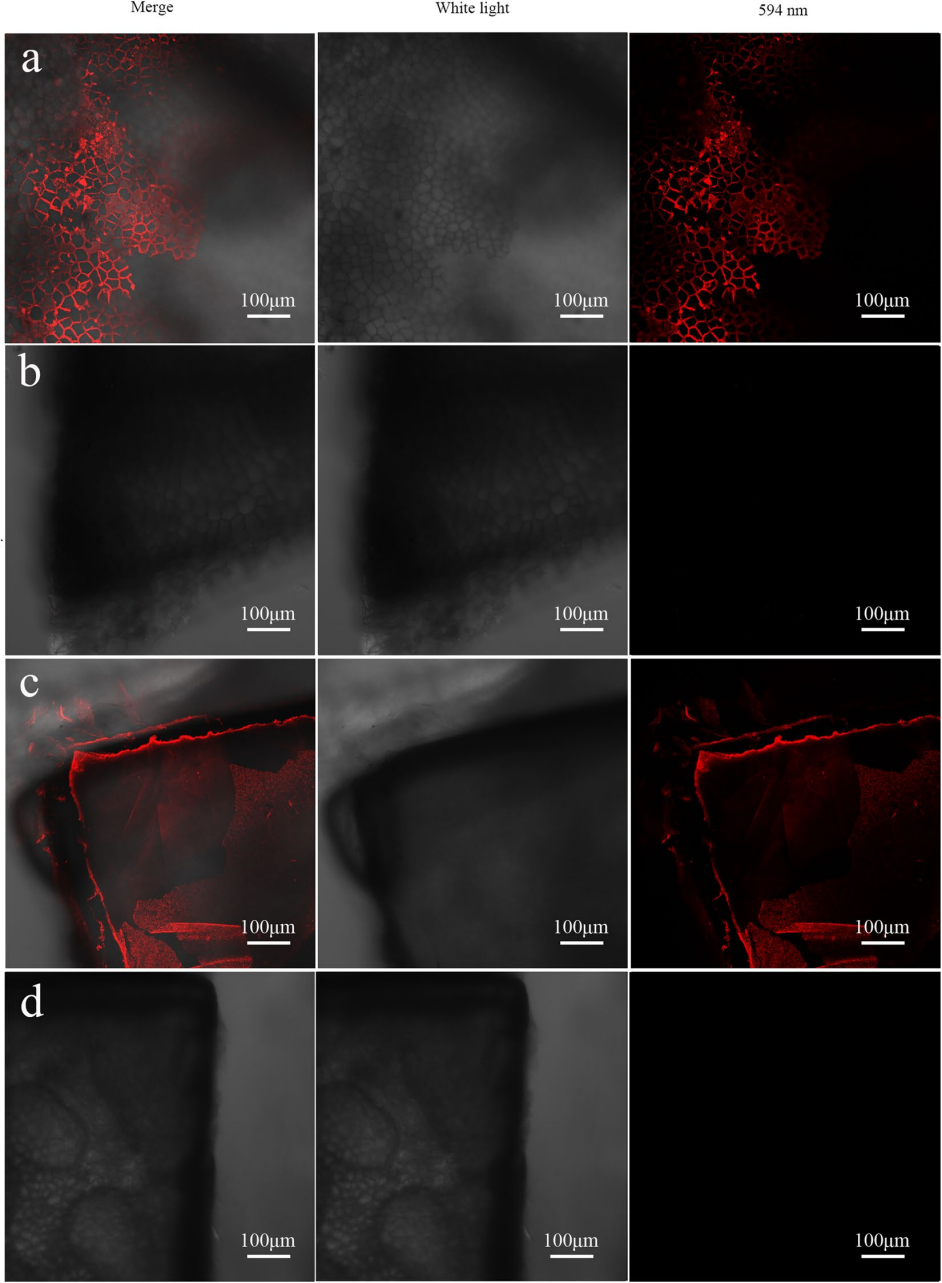
Immunofluorescence location of native Alv in the full-face of decalcified shells of *P*. *fucata*. (**a**) Images of decalcified prismatic layer with anti-rAlv polyclonal antibody as first antibody. (**b**) Images of decalcified prismatic layer with preimmune serum as first antibody. (**c**) Images of decalcified nacreous layer with anti-rAlv polyclonal antibody as first antibody. (**d**) Images of decalcified nacreous layer with preimmune serum as first antibody. white field, images observed under white light; 594 nm, images observed under 594 nm laser; merge, images merged by white field image and 594 nm image.

**Figure 6:**
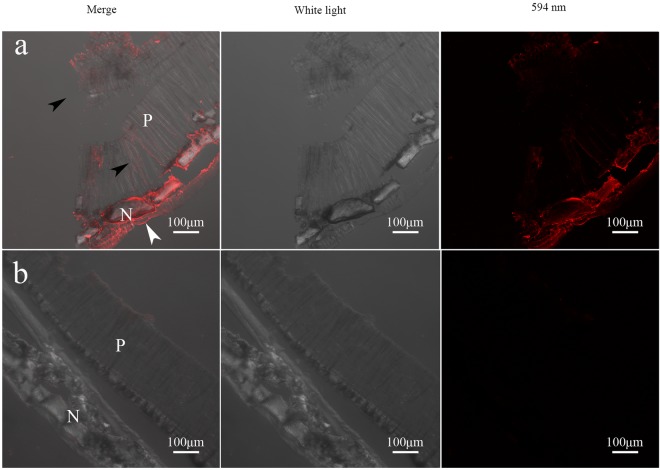
Immunofluorescence location of native Alv in the cross-section pieces of decalcified shell from *P*. *fucata*. (**a**) Images of cross-section pieces with anti-rAlv polyclonal antibody as first antibody. (**b**) Images of cross-section pieces with preimmune serum as first antibody. P, prismatic layer; and N, nacreous layer. The black arrows indicate the immunofluorescence location in the prismatic layer, and the white arrow indicates the immunofluorescence location in the nacreous layer.

**Figure 7:**
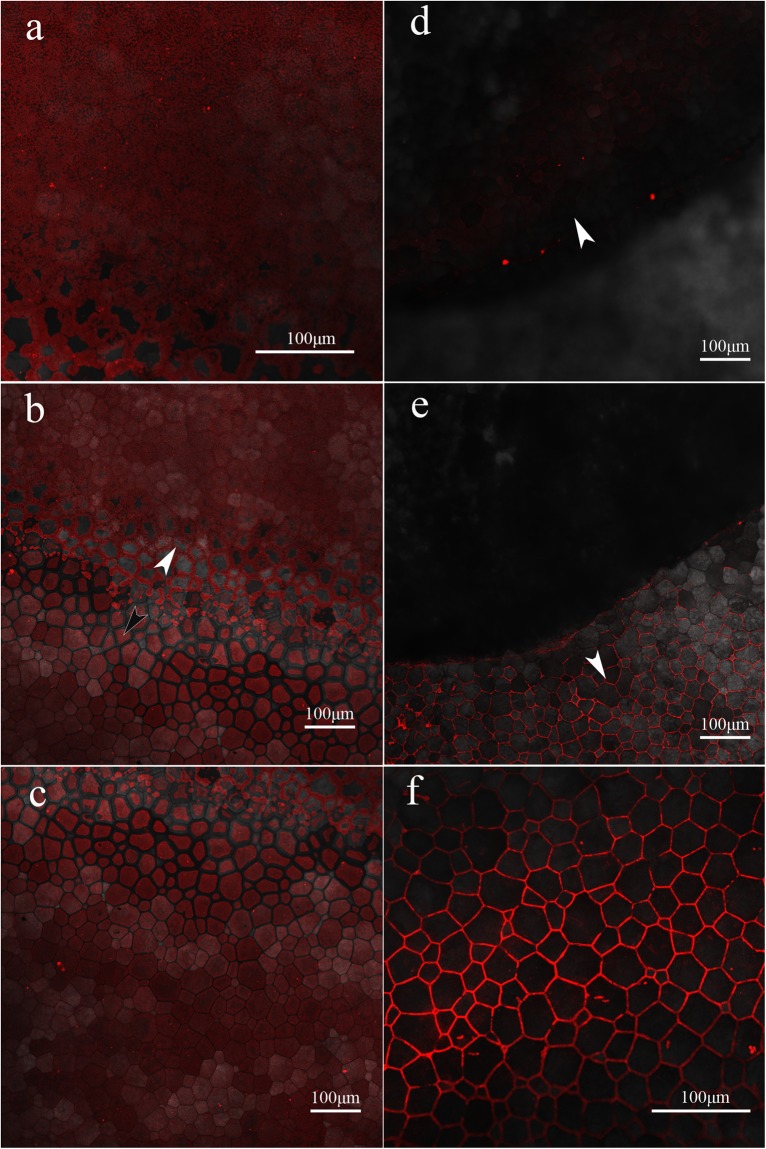
Immunofluorescence location of native Alv in the native shell from *P*. *fucata*. (**a**) Immunofluorescence image of nacreous layer. (**b**) Immunofluorescence image of border between nacreous layer and prismatic layer. The white arrow points to the nacreous layer, and the black arrow points to the prismatic layer around the border. (**c**) Immunofluorescence image of prismatic layer near the border. (**d**) Immunofluorescence image of inner prismatic layer far from the border. The white arrow indicates the inner prismatic layer. (**e**) Immunofluorescence image of outer prismatic layer far from the border. The white arrow indicates the outer prismatic layer. (**f**) Immunofluorescence image of outer prismatic layer near external rim of shell.

To reveal the microstructure location of native Alv in shell, undecalcified shells, full-faces of decalcified shells and frozen sections of the cross-section of decalcified shells were incubated by DyLight® 594-conjucted goat-anti-rabbit antibody^18,19^. As Fig. 5 shows, the immunofluorescence location of Alv in decalcified shells of *P*. *fucata* was existed in both the prismatic sheath and nacreous layer, which is consistent with the gene expression analysis and biochemical location^38,39^. To confirm the location of Alv in prismatic sheath and nacreous layer, we also explored cross-section of decalcified shells (Fig. 6). We found that Alv exist in both the decalcified prismatic (black arrows) and nacreous layer (white arrow). A small amount of background staining was visible in the control group but was much weaker than that of the experimental group. What’s more, we also observed the full-face of the non-demineralized prismatic layer and nacreous layer, which were only cleaned by sonicating. As Fig. 7 shows, the immunofluorescence location of Alv was existed in the prismatic and nacreous lamellae, which is consistent with the gene expression analyses in mantle and EISM location by Western blotting. A small amount of background staining was also visible in the control group but was much weaker than the experimental group. Surprisingly, around the boundary of the prismatic and nacreous layers, the fluorescence intensity of Alv protein is stronger and does not fill the surface of the lamellae, but rather the edge of the location is parallel to the margin of the lamellae. While the location of Alv in the inner prismatic layer which is away from the border of the prismatic and nacreous layers is uniform on the prismatic lamellae and prismatic sheath, and the fluorescence intensity is weaker than that of lamellae around the border. More dramatically, the Alv location in the outer and mature prismatic layer concentrates in the prismatic sheath.

## Discussion

The shell matrix protein of *P*. *fucata* was first obtained by classical fractionation and then identified by microarray in previous study. Alv, which exists in the prismatic layer of *P*. *margaritifera* and *P*. *maxima* with high homology, was first reported by Marie B *et al*.^26^. In 2015, Chuang Liu found that Alv also exists in the prismatic layer of *P*. *fucata* using a proteomic approach^27^. In Liu’s research, Alv was the third most abundant matrix protein in the prismatic layer. Currently, Alv is the first studied matrix protein that was obtained by a proteomic analysis. There are Alv homologous proteins in *P*. *maxima* and *P*. *margaritifera* with high conservation, indicating that the Alv protein might play important role in biomineralization. Although the function of VP repeated sequences found in the Alv protein is unclear, it may be related to arranging the repeated structure of polymers, such as chitin according to study before^40^. Additionally, the predicted secondary structure of the Alv protein is composed of almost 80% β-strand. The Rebers-Riddiford chitin binding motif^41,42^, which is the most spread motif in chitin-binding proteins, indicating that the chitin-binding domains of chitinases are characterized by forming β-strand structures^39,43^. The mantle was reported to be important for biomineralization of which the mantle pallial is responsible for the nacreous layer formation and the mantle edge contributes to the prismatic layer formation^6^. Real-time quantitative PCR result of Alv had highest expression in the mantle suggests the contribution of Alv to shell formation, which is consistent with the expression model of other matrix proteins. Expression of Alv was also detected in the outer epithelial cells of the outer fold (OF), outer epithelial cells of the middle fold (MF), and a part of the mantle pallial by *in situ* hybridization experiments, implying the functions of Alv in both the prismatic and nacreous layers^6^. To confirm confirm whether native Alv exists in both layers, western blot was executed, and the results showed that the native Alv protein exists in the EISM from both layers. It is reported that EISM proteins are responsible for the construction of the organic framework of the shell; therefore, it is logical to presume that Alv is a part of the frame component according to its location and amino acids composition^33,44^.

**Figure 8:**
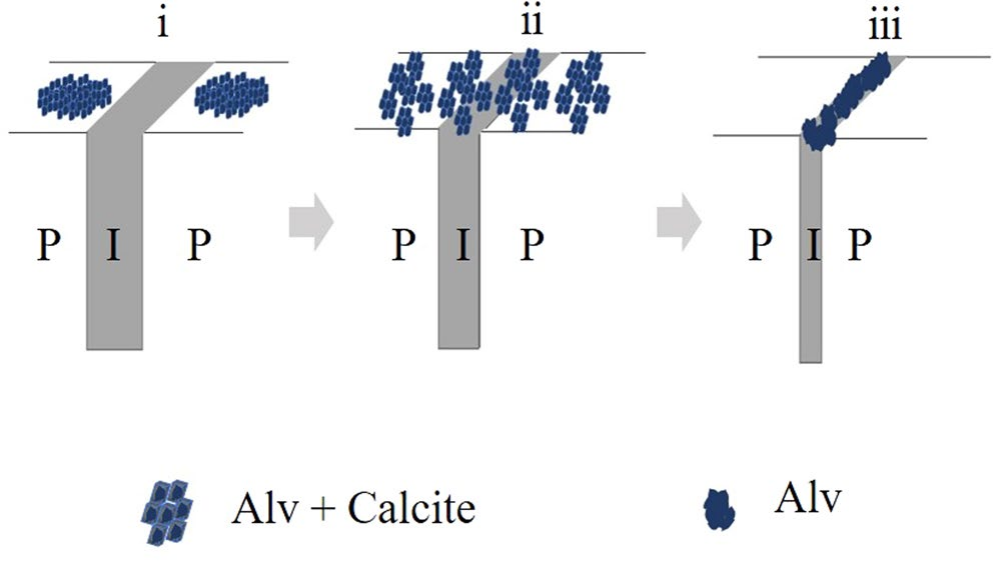
Schematic illustration of the proposed roles of Alv in the prismatic layer formation in *P*. *fucata*. Three stages from immature prism to mature prism. (**i**) Immature prism. Alv was secreted from mantle tissue and adhere at the immature prismatic layer to support nucleation sites and stimulate calcite crystallization. (**ii**) Transition prism. Alv was dispersed uniformly in second stage of prism formation as the calcite lamella grows. (**iii**) Mature prism. Alv was crushed by growing calcite and located in the in-between organic sheath exclusively. P, prism; N, nacre; I, in-between organic sheath.

The immunofluorescence location details of native Alv in the shell of *P*. *fucata* showed that native Alv was located in both layers. Before this study, details of matrix proteins in the whole shell had never been identified and characterized in mollusks. Hence, the detection of Alv in the whole shell could enrich matrix protein detection and the location study. The discovery of different immunofluorescence distributions of Alv in the inner and outer prismatic layers provides clues on the formation of the prismatic layer. Prior to this, scientists proposed many hypotheses about the relationship between the prismatic sheath and prismatic lamellae. According to our study, the distribution pattern supported Erben’s “crush” model, in which the sheath was crushed into the margin of the prismatic lamellae^45^. According to the finding of different location pattern in different prismatic layers, we assume the Alv protein was first secreted onto the center of the lamellae to offer nucleation sites and was then distributed following prismatic lamellae growth. As the prismatic layer grew, the Alv protein was squeezed and was distributed into the prismatic sheath (Fig. 8). This presume could be supported by Erben’s model and Checa’s model and could enrich the assembly of macromolecules and the formation of the prismatic layer^45,46^. However, the distribution of more matrix proteins needs to be studied to either confirm the theory or to modify the theory, as many questions, such as why sheaths have the same width, cannot be explained.

Until now, only two matrix proteins of *P*. *fucata*, KRMP-3^18^ and MSI7^28^ were suggested to have dual roles in aragonite and calcite formation *in vitro*, which have not been proven *in vivo*. Most matrix proteins only have functions in one layer. Alv are thought to be involved in formation of prism and nacre according to polyclonal antibody injection assay. The inhibition of functions of Alv in *P*. *fucata* led to opposite results of a destroyed prismatic layer and an overgrown nacreous layer which is rare in functions of matrix proteins and need to be further studied. According to the location of Alv in different prismatic layers in shell, three stages of prismatic layer were divided. At the first stage of prism formation, Alv was secreted from mantle tissue and adhere at the prismatic layer to influence calcite crystallization. Along with the growth of calcite lamella, the Alv was dispersed uniformly in second stage of prism formation. At mature stage of prism, Alv was crushed by growing calcite and distributed in the in-between organic sheath exclusively.

In conclusion, Alv, as a matrix protein exists in prism and nacre of *P*. *fucata*, plays important roles in shell formation. In this study, we cloned full-length cDNA sequence of *Alv* and explored the function of Alv *in vivo*. What’s more, the finding of different location of Alv in prismatic layer provides insights into the formation of the prismatic layer of *P*. *fucata*.

## Electronic supplementary material

supporting information
