# Supplementary material for: Cloning, characterization and functional analysis of an Alveoline-like protein in the shell of Pinctada fucata
Source: Sci Rep. 2018 Aug 16;8:12258. doi: 10.1038/s41598-018-29743-6 (PMC6095885; doi:10.1038/s41598-018-29743-6)
Supplement: Supplementary file 1 — supporting information [file 41598_2018_29743_MOESM1_ESM.docx]

**Supplementary information**

**Cloning, characterization and functional analysis of an Alveoline-like protein in the shell of *Pinctada fucata***

Jingjing Kong^1^, Chuang Liu^1,2^, Tianpeng Wang^1^, Dong Yang^1^, Yi Yan^1^, Yan Chen^1^, Yangjia Liu^1^, Jingliang Huang^1^, Guilan Zheng^1^, Liping Xie^1^, and Rongqing Zhang^1,2^*

1 Protein Science Laboratory of the Ministry of Education, School of Life Sciences, School of Life Sciences, Tsinghua University, Beijing 100084 China;

2 Department of Biotechnology and Biomedicine, Yangtze Delta Region Institute of Tsinghua University, Jiaxing, Zhejiang Province, 314006, China

*To whom correspondence may be addressed. E-mail: rqzhang@mail.tsinghua.edu.cn. Tele: +86-010-62772630.


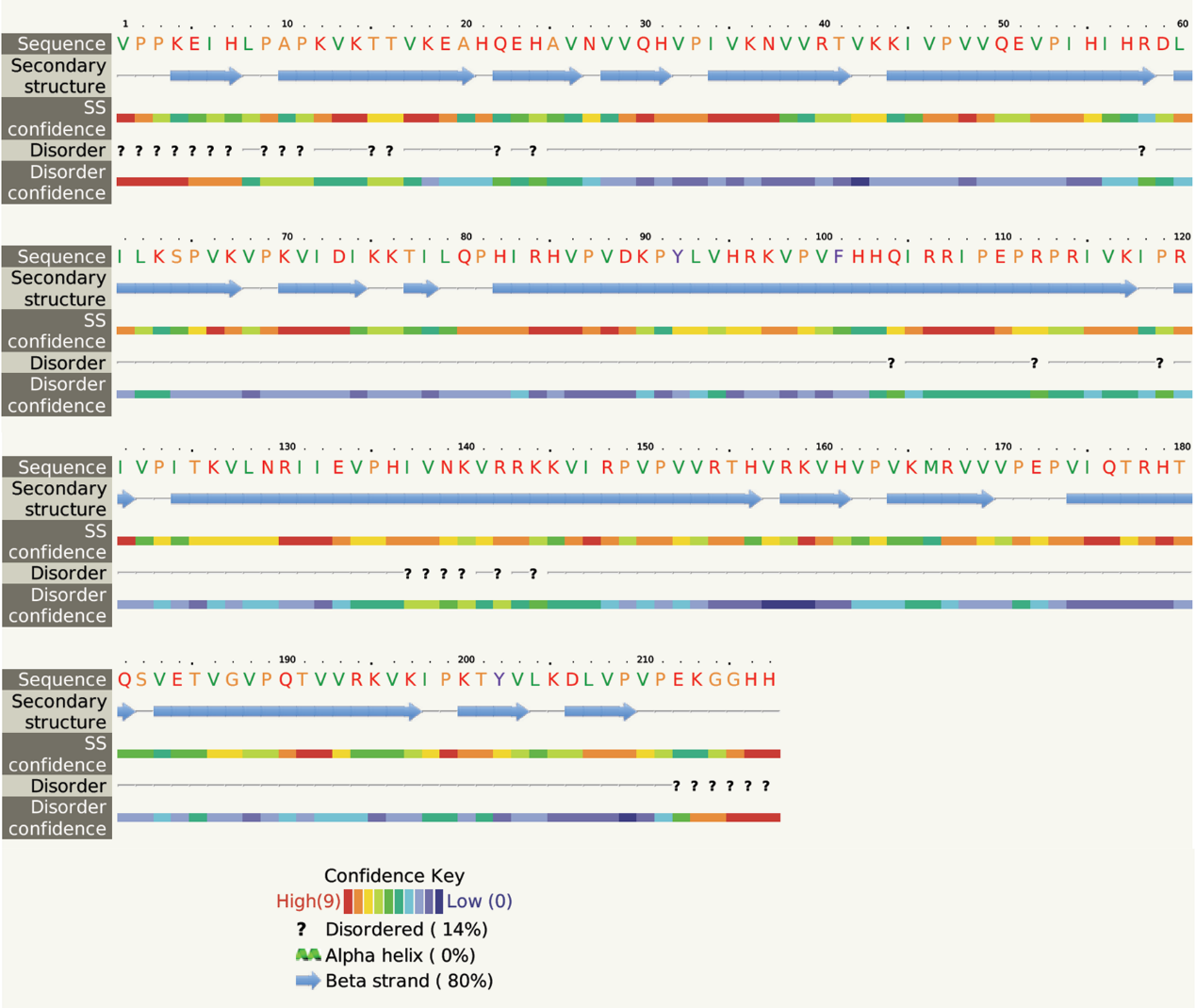


**Figure S1. The secondary structure of the Alv protein without signal peptide predicted by the Phyre2 website.**


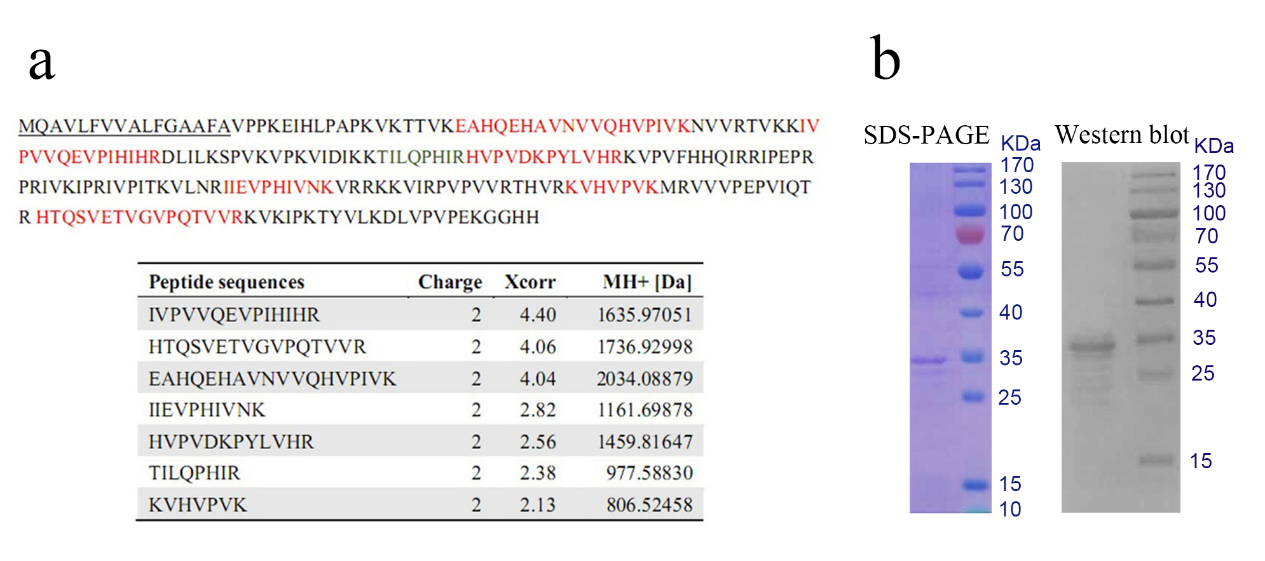


**Figure S2.** a. LC-MS/MS analyses of Alv. The signal peptide is underlined, the matched MS/MS peptides are marked red or green, and the table lists the corresponding parameters of peptides. b. SDS-PAGE and Western blot of rAlv purification with anti-Alv polyclonal antibody as first antibody. Please refer to Fig. S5c to get the primary gel and blot pictures.


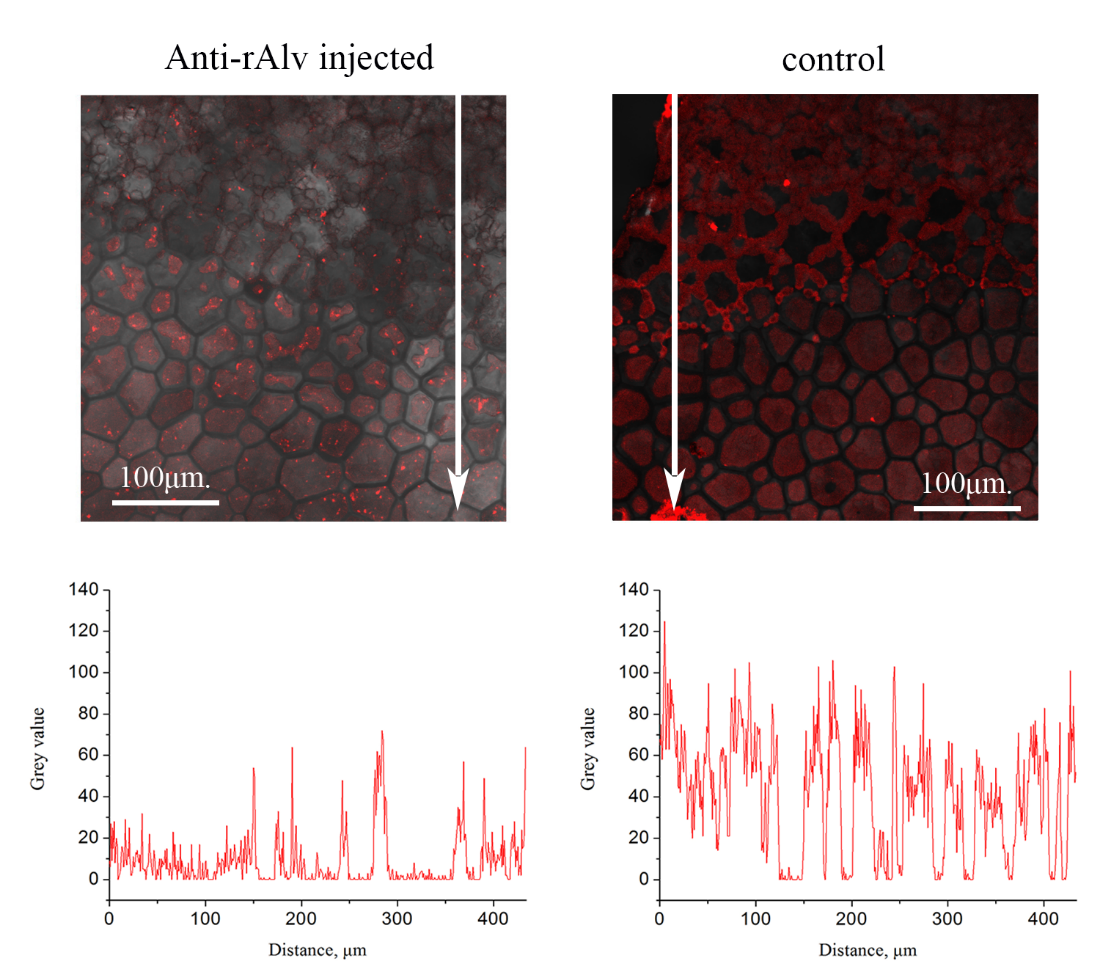


**Figure S3. Immunofluorescence intensity of the blue lines of shells from the anti-rAlv injected group and control group.** The white arrows point to the *x*-axis direction.


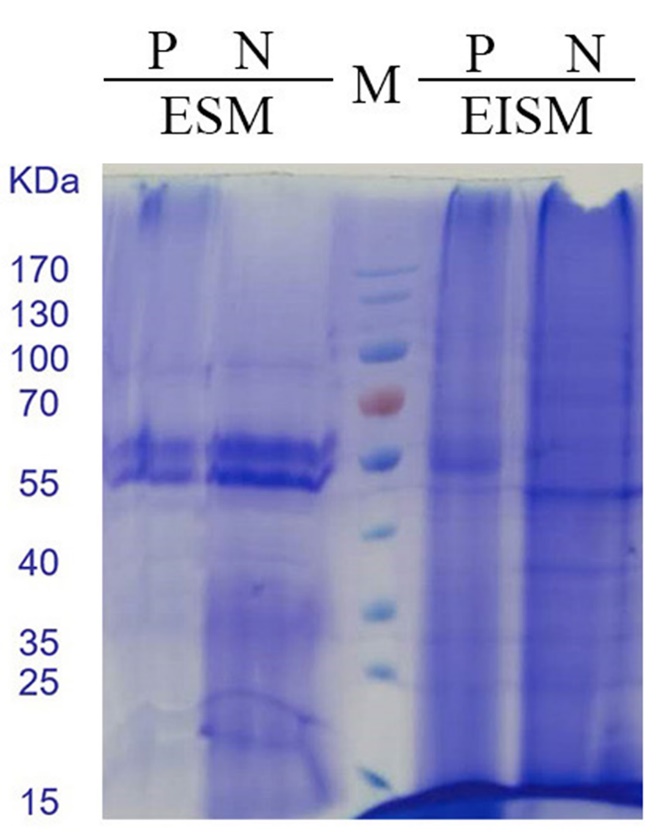


**Figure S4.** **SDS-PAGE of shell matrix protein.** ESM, EDTA-soluble matrix; EISM, EDTA-insoluble matrix; P, prismatic layer; N, nacreous layer; and M, marker. Please refer to Fig. S5d to get the primary gel pictures.


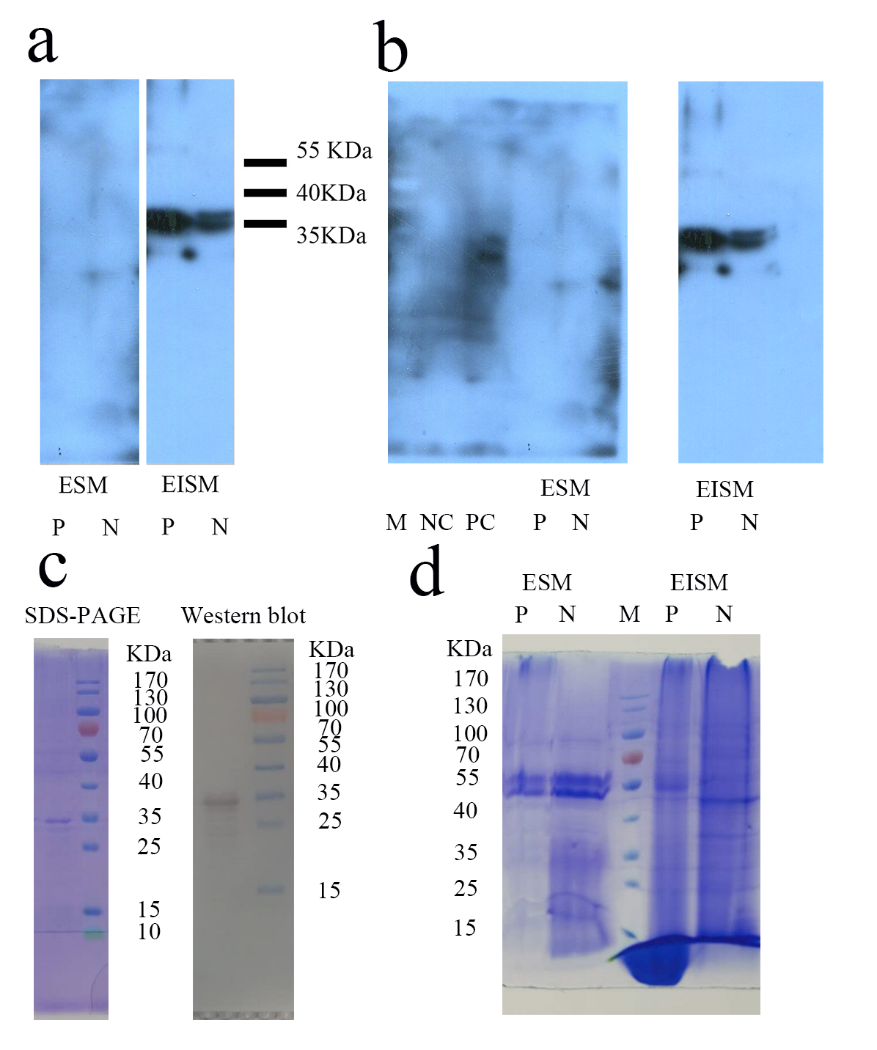


**Figure S5.** **Full-length and original images of gels and blots in manuscript.** a. original image of Fig. 4d (Western blotting analyses of Alv in a matrix protein. ESM, EDTA-soluble matrix; EISM, EDTA-insoluble matrix; P, prismatic layer; and N, nacreous layer.). b. Full-length image of Fig. 4d (Western blotting analyses of Alv in a matrix protein. M, marker; NC, negative control, non-induced recombinant *E. coli*; PC, positive control, induced recombinant *E. coli*; ESM, EDTA-soluble matrix; EISM, EDTA-insoluble matrix; P, prismatic layer; and N, nacreous layer.). c. Full-length and original image of Fig. S2b (SDS-PAGE and Western blot of rAlv purification with anti-Alv polyclonal antibody as first antibody.). d. Full-length image of Fig. S4 (SDS-PAGE of shell matrix protein. ESM, EDTA-soluble matrix; EISM, EDTA-insoluble matrix; P, prismatic layer; N, nacreous layer; and M, marker.)
